# *Phlebotomus perniciosus* Recombinant Salivary Proteins Polarize Murine Macrophages Toward the Anti-Inflammatory Phenotype

**DOI:** 10.3389/fcimb.2020.00427

**Published:** 2020-08-24

**Authors:** Petra Sumova, Nikola Polanska, Tereza Lestinova, Tatiana Spitzova, Barbora Kalouskova, Ondrej Vanek, Petr Volf, Iva Rohousova

**Affiliations:** ^1^Laboratory of Vector Biology, Department of Parasitology, Faculty of Science, Charles University, Prague, Czechia; ^2^Laboratory of Structural Biochemistry of Immune Recognition, Department of Biochemistry, Faculty of Science, Charles University, Prague, Czechia

**Keywords:** *Phlebotomus*, sand fly saliva, yellow-related proteins, apyrase, macrophage polarization, immunogenicity

## Abstract

*Phlebotomus perniciosus* (Diptera: Phlebotominae) is a medically and veterinary important insect vector. It transmits the unicellular parasite *Leishmania infantum* that multiplies intracellularly in macrophages causing life-threatening visceral diseases. *Leishmania* establishment in the vertebrate host is substantially influenced by immunomodulatory properties of vector saliva that are obligatorily co-injected into the feeding site. The repertoire of *P. perniciosus* salivary molecules has already been revealed and, subsequently, several salivary proteins have been expressed. However, their immunogenic properties have never been studied. In our study, we tested three *P. perniciosus* recombinant salivary proteins—an apyrase rSP01 and yellow-related proteins rSP03 and rSP03B—and showed their anti-inflammatory nature on the murine bone-marrow derived macrophages. Even in the presence of pro-inflammatory stimuli (IFN-γ and bacterial lipopolysaccharide, LPS), all three recombinant proteins inhibited nitric oxide production. Moreover, rSP03 seems to have a very strong anti-inflammatory effect since it enhanced arginase activity, increased the production of IL-10, and inhibited the production of TNF-α even in macrophages stimulated with IFN-γ and LPS. These results suggest that *P. perniciosus* apyrase and yellow-related proteins may serve as enhancing factors in sand fly saliva, facilitating the development of *Leishmania* infection along with their anti-haemostatic properties. Additionally, rSP03 and rSP03B did not elicit the delayed-type hypersensitivity response in mice pre-exposed to *P. perniciosus* bites (measured as visible skin reaction). The results of our study may help to understand the potential function of recombinant's native counterparts and their role in *Leishmania* transmission and establishment within the host.

## Introduction

*Phlebotomus perniciosus* (Diptera: Phlebotominae) is a sand fly species distributed in western part of Mediterranean basin. This species is medically and veterinary important as a vector of phleboviruses (e.g., Toscana virus) and, most importantly, as a vector of *Leishmania infantum* (Kinetoplastea: Trypanosomatida), a protozoan intracellular parasite that causes zoonotic human leishmaniasis with canids as the main reservoirs (Maroli et al., 2013).

Sand fly females feed on blood to gain enough nutrients for eggs development. During probing and sucking, the female obligatorily deposits a mixture of salivary molecules into the host skin. Besides being anti-haemostatic, the sand fly salivary molecules are also immunogenic, eliciting both humoral and cellular immune responses in repeatedly bitten host. Such anti-saliva immune response can be employed in leishmaniasis control. The anti-saliva antibody response can be utilized as the tool to screen hosts for the sand fly exposure at the individual level (e.g., in vector control programs), while the anti-saliva cellular immune response has been shown to protect the host against severe leishmaniasis (Lestinova et al., 2017).

The complexity of sand fly salivary proteins has already been revealed for several sand fly species (Coutinho-Abreu and Valenzuela, 2018; Oliveira et al., 2020; Polanska et al., 2020), including *P. perniciosus* (Anderson et al., 2006; Martin-Martin et al., 2013). Several salivary proteins from this sand fly species have been expressed and further tested primarily as markers of exposure (Lestinova et al., 2017; Velez et al., 2018; Willen et al., 2018, 2019; Risueno et al., 2019; Burnham et al., 2020; Maia et al., 2020). However, the functional studies of *P. perniciosus* salivary recombinant proteins are very limited (Sumova et al., 2019), and so far there is no study on their immunomodulatory properties.

In our study, we functionally tested recombinant salivary proteins of *P. perniciosus*, focusing on macrophages as the key cells in leishmaniasis. Macrophages are playing a dual role during *Leishmania* infection. They serve as the main host cell for *Leishmania* parasites as well as the key immune cell controlling the infection (Tomiotto-Pellissier et al., 2018). There are two major macrophage populations with different functions, classically activated M1 macrophages and alternatively activated M2 macrophages. M1 macrophages are activated in the presence of microbial substrates (e.g., bacterial lipopolysaccharide, LPS) and pro-inflammatory cytokines such as IFN-γ and TNF-α. Upon stimulation, they secrete high levels of pro-inflammatory cytokines (e.g., TNF-α, IL-1, IL-6, IL-12) and express the inducible nitric oxide synthetase (iNOS) responsible for the production of microbicide nitric oxide that kills intracellular *Leishmania* and thus controls the infection (Shapouri-Moghaddam et al., 2018; Tomiotto-Pellissier et al., 2018). The latter one, M2 macrophages (also referred as tissue-repairing macrophages), are activated by anti-inflammatory cytokines such as IL-4 and IL-13 toward the expression of the enzyme arginase and high production of IL-10 and TGF-β (Shapouri-Moghaddam et al., 2018). The products of arginase (L-ornithine and its metabolites) then favor *Leishmania* survival and multiplication (Tomiotto-Pellissier et al., 2018). With high functional plasticity, macrophages can switch from one phenotype to another, responding to the changes in the ongoing immune response (Shapouri-Moghaddam et al., 2018).

We focused on the salivary proteins of two abundant protein families; the yellow-related proteins (YRPs) and the apyrases. We compared the effect of these recombinant proteins on the murine macrophages activated either by classical or alternative pathway by measuring nitrite as a marker of iNOS activity and urea as a marker of arginase activity. Additionally, we measured the levels of corresponding secreted cytokines for the recombinant protein with the most pronounced immunomodulatory effect and its ability to elicit delayed-type hypersensitivity immune response in repeatedly bitten mice. The results of our study may help to understand the possible function of their native counterparts.

## Materials and Methods

### Animals and Ethics Statement

Female mice, strain BALB/c and SKH, were purchased from Velaz (the Czech Republic). The mice were used for the experiments at 7–10 weeks of age. Animals were maintained and handled in the animal facility of the Charles University in accordance with the institutional guidelines and Czech legislation (Act No. 246/1992 and 359/2012 coll. on Protection of Animals against Cruelty in present statutes at large), which includes all relevant European Union and international guidelines for experimental animals. The experiments were approved by the Committee on Ethics of Laboratory Experiments of the Charles University and were performed under the permission of the Ministry of the Environment of the Czech Republic (number: MSMT-10270/2015-6) and the Certificate of Competency (number: CZ 02451) approved by the Ministry of Agriculture of the Czech Republic.

### Sand Flies and Salivary Gland Dissection

The colony of *P. perniciosus* (originating from Murcia, Spain) was reared under the standard conditions as described in Volf and Volfova (2011). Salivary glands for macrophage stimulation were dissected from 3- to 5-day-old female sand flies, placed into RPMI-1640 medium (Sigma) and stored at −20°C until needed. Salivary glands for delayed-type hypersensitivity test were dissected into the phosphate-buffered saline (PBS; pH 7.2). Before use, the salivary glands were disrupted by three freeze-thaw cycles to prepare the salivary gland homogenate (SGH). All preparations of saliva were sterilized (5 min, 9,300 × g) through 0.22 μm pore centrifugal filters (Millipore) before use in *in vitro* experiments.

### Recombinant Proteins

Two yellow-related proteins (rSP03 and rSP03B, Gen Bank ACCN ABA43049 and ABA43050, respectively) and apyrase (rSP01, Gen Bank ACCN ABB00906) derived from *P. perniciosus* salivary proteins were expressed in a human cell line. All proteins were transiently expressed in HEK293S GnTI^−^ cells (ATCC CRL-3022) as previously described (Blaha et al., 2015). Proteins rSP03 and rSP03B were prepared as described in Sumova et al. (2019). Apyrase rSP01 was expressed in a same way as protein rSP03B. Briefly, transiently transfected HEK293S GnTI^−^ cells were grown for five to seven days before harvesting the culture medium. The histidine-tagged product was then recovered by IMAC chromatography on HiTrap TALON crude columns (GE Healthcare) and further purified by SEC on Superdex 200 10/300 GL column (GE Healthcare). Finally, the protein identity was verified by mass spectrometry (BIOCEV, the Czech Republic).

### Bone Marrow Macrophages Culture

Bone marrow was obtained by flushing of tibias and femurs of euthanized BALB/c mice. The differentiation from the bone marrow precursor cells to the bone marrow-derived macrophages proceeded for 7–9 days in the sterile polystyrene Petri dishes (Thermo Fisher) in the presence of L929 fibroblast cell culture supernatant (20% in RPMI-1640 medium), serving as a source of macrophage colony-stimulating factor (M-CSF), at 37°C with 5% CO_2_. Later, after differentiation, the macrophages were cultured in RPMI-1640 medium (Sigma) containing 10% FBS, 1% penicillin-streptomycin (Sigma), 2 mM of L-glutamine (Sigma) and 0.05 mM of β-mercaptoethanol (referred as a complete medium) at 37°C with 5% CO_2_.

### Bone Marrow Macrophages Stimulation

The sessile bone marrow-derived macrophages (BMMF) were released from the Petri dish with trypsin-EDTA solution (Sigma), washed in 0.9% saline solution and seeded into the 96-wells plates (Costar) at a density of 1 × 10^6^ cells/ml (5 × 10^4^ cells/well). Consequently, the macrophages were co-incubated with *P. perniciosus* SGH (Per-SGH) in the amount equivalent to 0.5 salivary gland per well or with 0.2 μg of one of the recombinant proteins (rSP03, rSP03B, or rSP01) per well. The concentration of Per-SGH and of recombinant proteins has been selected based on our previous studies (Rohousova et al., 2005; Drahota et al., 2009) and on the preliminary experiments (data not shown). Control cells were incubated solely in complete RPMI-1640. Two hours later, cells were stimulated with a combination of IFN-γ (50 U/ml, AbD SEROTEC) and LPS (0.5 μg/ml, Sigma) (referred as classically stimulated macrophages), with IL-4 (25 ng/ml, eBioscience) (referred as alternatively stimulated macrophages), or left unstimulated. After 72 h of incubation, the supernatant and cell lysate were used for the nitrite and urea analysis, respectively. For the cytokine analysis, macrophages were incubated for 24 h and the collected supernatant was stored in −80°C until needed. In all experiments, samples were analyzed in pentaplicates. Six independent experiments were performed with 6 mice in total.

### Nitrite Analysis to Measure iNOS Activity

The macrophage iNOS activity was analyzed by measuring the accumulation of nitric oxide in the culture supernatant over a 72 h period. Since the nitric oxide is a short lived free radical, we measured its product, the nitrite ion, using a Griess test in a microplate assay. A total of 100 μl of the culture supernatant was mixed with 50 μl of 60 mM sulfanilamide in 2.5% phosphoric acid and incubated at room temperature in dark for 5 min. Thereafter, 50 μl of 12 mM N-1-naphthylethylenediamine dihydrochloride in 2.5% phosphoric acid was added and incubated in dark for additional 5 min. The absorbance was read at 550 nm using the microplate reader (Tecan Infinite M200). The nitrite concentration was determined using a sodium nitrite as a standard in the range of 12.5–100 μM.

### Urea Analysis to Measure Arginase Activity

Arginase activity was analyzed in the lysate of macrophages by measuring the conversion of L-arginine to urea as previously described (Kropf et al., 2007) with slight modifications. Cells were lysed with a solution of Tris–HCl in combination with protease inhibitors (Complete Mini, Roche) and Triton X-100. The enzyme was then activated in the presence of 10 mM MnCl_2_ by heating and arginine hydrolysis was carried out by incubation of the activated enzyme with 0.5 M arginine (Sigma–Aldrich) at 37°C with 5% CO_2_ for 120 min. The reaction was stopped with 400 μl of solution containing H_2_SO_4_, H_3_PO_4_ and distilled water (at ratio of 1:3:7). Color reaction was developed in the presence of 20 μl 550 mM α- isonitrosopropiophenone (dissolved in 100% ethanol) after incubation at 100°C for 45 min. Samples were then transferred to microplates and absorbance was read at 540 nm using the microplate reader (Tecan Infinite M200). Urea concentration was determined using urea as a standard in the range of 0.004–0.6 mg/ml.

### Detection of Cytokine Production

The production of IL-10 and TNF-α was determined by sandwich enzyme-linked immunosorbent assays (ELISA) using ELISA MAX^TM^ Standard Set kits (BioLegend) according to the manufacturer's instruction with the following modification. Undiluted supernatant was used for determination of IL-10 production, while TNF-α was measured in supernatant diluted twenty-times in RPMI-1640 medium (Sigma). Absorbance was measured using a microplate reader (Tecan Infinite M200).

### Skin Test to Measure Delayed-Type Hypersensitivity

The skin test was performed on two females of SKH mice. Mice were exposed to *P. perniciosus* bites, once per week, repeated three times with a 1-week interval between them during three consecutive weeks, with 50–100 bloodfed females per mouse per exposure. Only the dorsal part of the mouse body was available for bloodfeeding. Fourteen days after the last exposure, mice were anesthetized and challenged intradermally on the ventral part of the body with 0.2 μg (in a volume of 12.5 μl) of rSP03 and rSP03B separated from each other as depicted on the **Figure 3B**. The same volume of Per-SGH (equivalent to 0.5 gland) and PBS were used as positive and negative controls, respectively. The delayed-type hypersensitivity (DTH) reaction was measured 48 h post injection as the diameter of erythema.

### Endotoxin Test

The level of endotoxin (bacterial LPS) in SGH, recombinant proteins, *Leishmania* and bone marrow macrophages cultures was quantified by ToxinSensor^TM^ Chromogenic LAL Endotoxin Assay Kit (GenScript) according to the manufacturer instructions. Endotoxin levels were measured in the unstimulated macrophage culture and in all stimulants separately. In all preparations, the concentration of endotoxin was lower than 0.1 EU/ml, a level below the limit to activate the immune cells (Schwarz et al., 2014).

### Statistical Analysis

Statistical analyses were carried out using the R software (version 3.6.3, downloaded from http://www.r-project.org). The “nlme” package was used to develop the multilevel regression models to investigate relationship between nitrite concentration, urea concentration and cytokine production as the continuous dependent variables, and exposure of macrophages to saliva or recombinant proteins as the categorical predictor variables. This relationship was examined separately for the macrophages incubated alone in complete RPMI-1640, stimulated with IL-4, and stimulated with a combination of IFN-γ and LPS. Continuous dependent variables with exception of cytokine production were transformed using ln(x+1) formula. *P* < 0.05 indicated statistical significance. Because the samples were analyzed in pentaplicates, we considered the correlation between repeated measurements within the individual mouse.

The fold-change values were calculated as a ratio between the value for cells incubated with salivary proteins (either Per-SGH, rSP01, rSP03, or rSP03B) divided by the mean value of the control cells (marked as neg in figures) treated with the same macrophage stimulus (IFN+LPS, IL-4, or unstimulated).

## Results

### The Effect of Salivary Proteins on Arginase Activity

**Figure 1:**
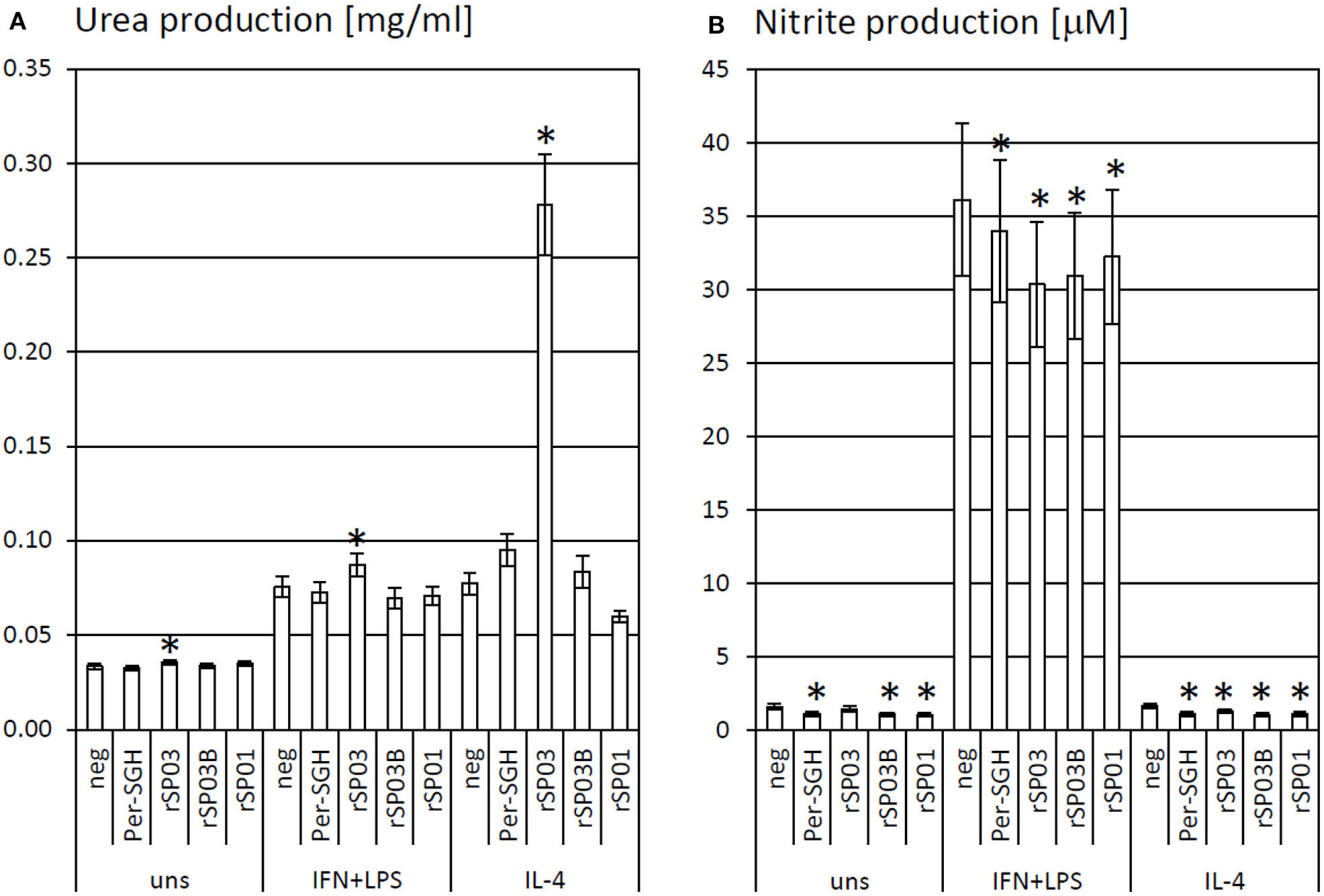
The effect of *Phlebotomus perniciosus* salivary proteins on urea and nitrite production. Bone marrow-derived macrophages of BALB/c mice were incubated with salivary gland homogenate of *Phlebotomus perniciosus* (Per-SGH), or with the following *P. perniciosus* recombinant proteins: Yellow-related proteins rSP03 and rSP03B, or Apyrase rSP01. The cells were stimulated with either IL-4 or a combination of IFN-γ and bacterial lipopolysaccharide (IFN+LPS) and subsequently tested for urea **(A)** or nitrite **(B)** production as markers of arginase and iNOS activity, respectively. Unstimulated (uns) and negative (neg) controls were included. The experiment was performed in six biological replicates (6 mice). The data are presented as mean ± standard error, (*) denotes statistical significance at *p* < 0.05 compared to the neg control of the appropriate stimulant.

Recombinant yellow-related proteins rSP03 and rSP03B, apyrase rSP01, and crude *P. perniciosus* SGH (Per-SGH) were incubated with the bone marrow-derived macrophages to measure the urea production (Figure 1A) as a marker of arginase activity that is typical for tissue-repairing macrophages. The most pronounced effect has been observed with rSP03 (Figure 1A), which significantly increased urea production regardless of the macrophages' stimulation; the highest production, 3.6-fold the negative control value, was observed in the cells stimulated with IL-4. No significant changes were observed after incubation with rSP03B, rSP01, nor with Per-SGH.

### The Effect of Salivary Proteins on iNOS Activity

rSP03, rSP03B, rSP01, and crude Per-SGH were incubated with BMMF to measure nitrite production (Figure 1B) as a marker of iNOS activity that is typical for pro-inflammatory macrophages. All tested proteins inhibited the production of NO, regardless of the macrophages' stimulation, and significantly in majority of combinations (Figure 1B). The most pronounced effect has been observed in the unstimulated and IL-4-stimulated macrophages incubated with Per-SGH (0.71 and 0.68-fold, respectively), followed by rSP03B (0.68 and 0.64-fold, respectively) and rSP01 (0.66 and 0.66-fold, respectively). All antigens significantly decreased the iNOS activity also in IFN-γ and LPS stimulated macrophages, the most pronounced effect was detected for rSP03 (0.84-fold the negative control value).

### The Effect of rSP03 on Cytokine Production

**Figure 2:**
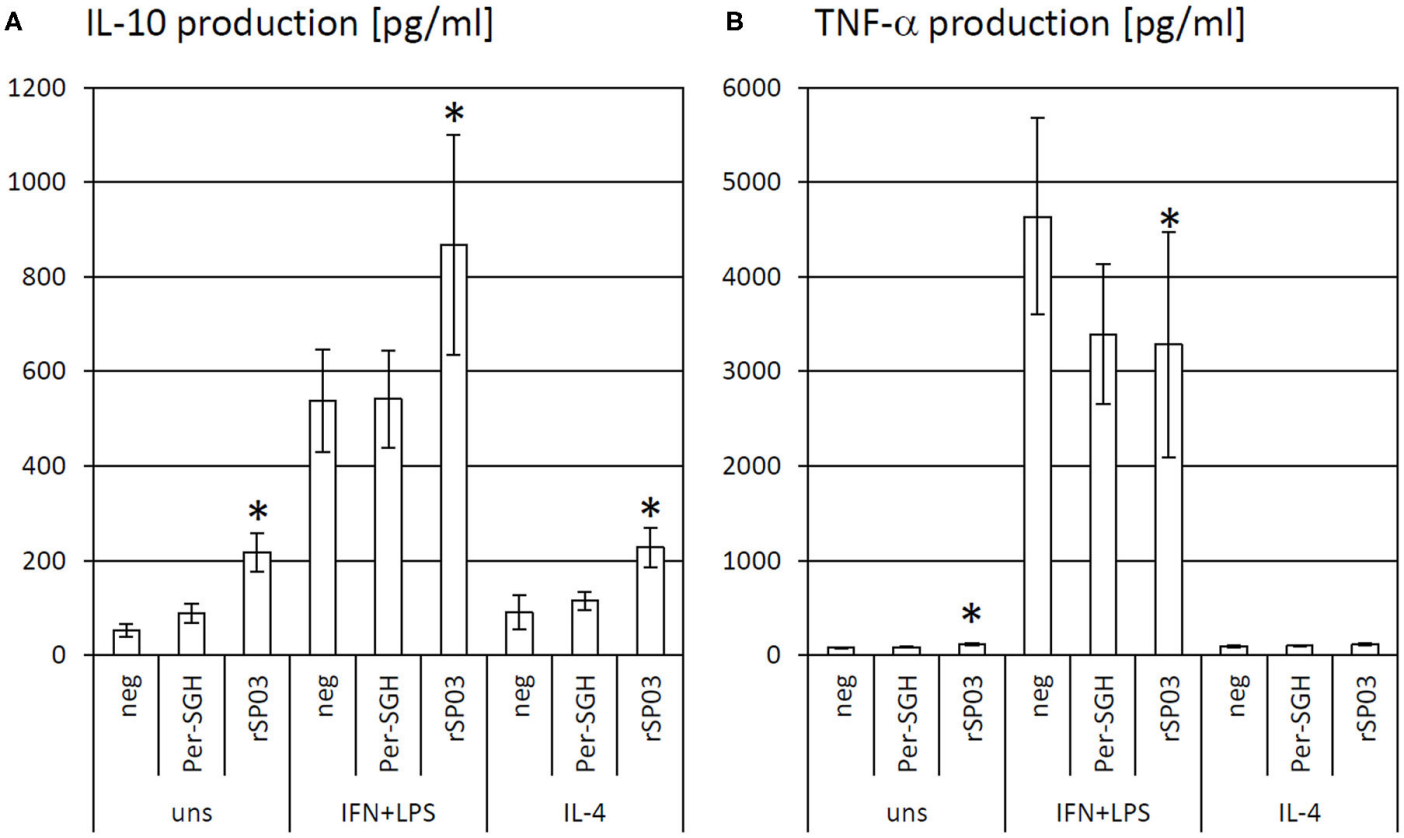
The effect of *Phlebotomus perniciosus* rSP03 on cytokine production. Bone marrow-derived macrophages of BALB/c mice were incubated with salivary gland homogenate of *Phlebotomus perniciosus* (Per-SGH) or with *P. perniciosus* recombinant Yellow-related protein rSP03. The cells were stimulated with either IL-4 or a combination of IFN-γ and bacterial lipopolysaccharide (IFN+LPS) and the supernatant was subsequently tested for the production of IL-10 **(A)** and TNF-α **(B)**. Unstimulated (uns) and negative (neg) controls were included. The experiment was performed in six biological replicates (6 mice). The data are presented as mean ± standard error, (*) denotes statistical significance at *p* < 0.05 compared to the neg control of the appropriate stimulant.

As the protein rSP03 had the most pronounced effect on macrophages' activities, its effect on cytokine production of differentially stimulated macrophages was also evaluated. rSP03 and crude Per-SGH were incubated with bone marrow-derived macrophages to measure the production of anti-inflammatory cytokine IL-10 (Figure 2A) and pro-inflammatory cytokine TNF-α (Figure 2B). rSP03 markedly increased IL-10 production in unstimulated macrophages (4.18-fold), as well as in macrophages stimulated either with IL-4 (2.52-fold) or a combination of IFN-γ and LPS (1.62-fold). rSP03 also significantly increased TNF-α production in unstimulated cells (1.47-fold), and decreased production of TNF-α in IFN-γ and LPS-stimulated macrophages (0.71-fold). Per-SGH followed similar trends as rSP03 but its effect was less prominent and statistically insignificant.

### Intradermal Skin Test With rSP03

**Figure 3:**
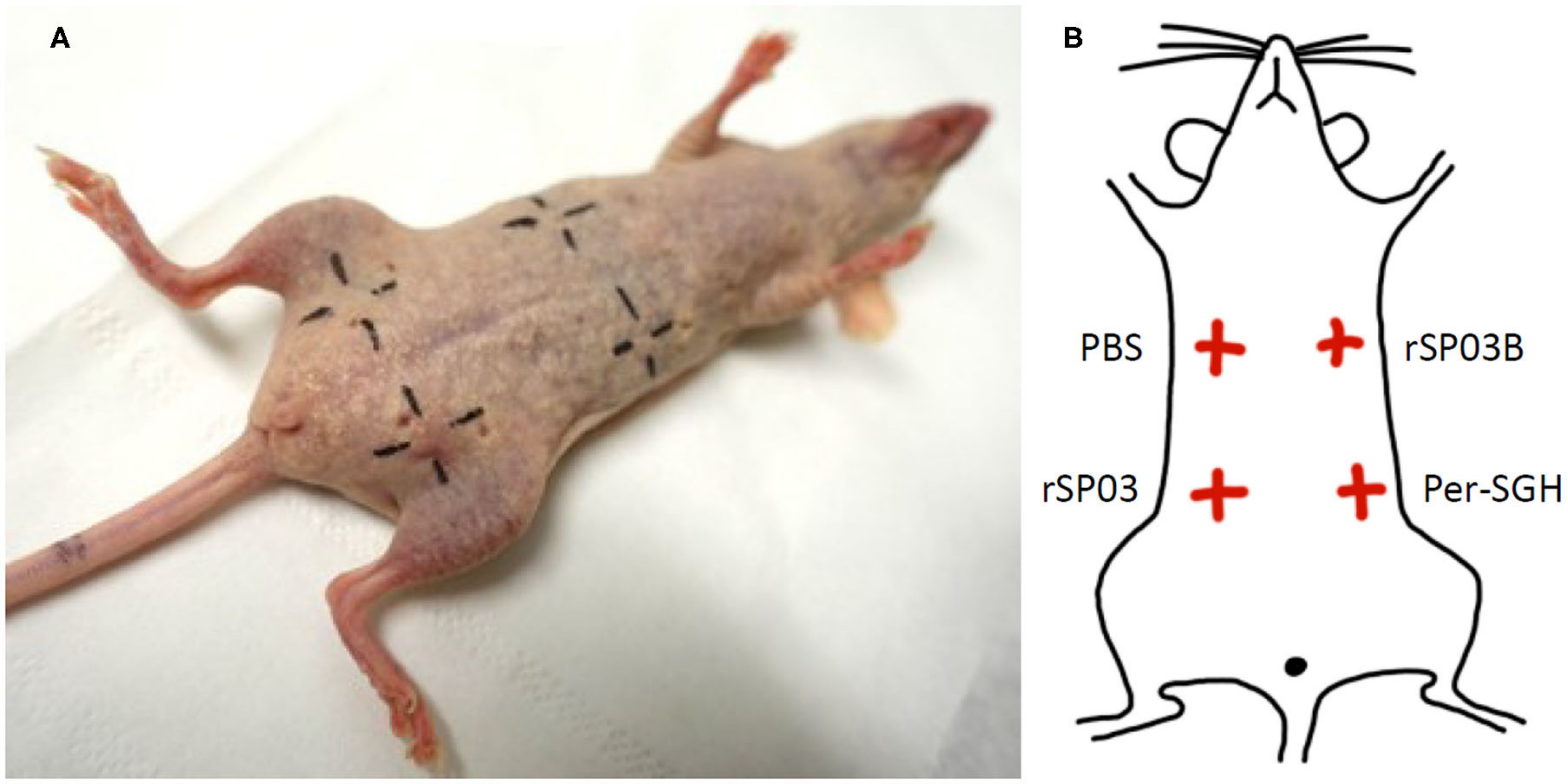
Intradermal skin test with *Phlebotomus perniciosus* recombinant salivary proteins rSP03 and rSP03B. SKH mice were repeatedly exposed to *Phlebotomus perniciosus* bites. Two weeks after the last exposure, mice were challenged intradermally with *P. perniciosus* recombinant Yellow-related proteins rSP03 and rSP03B, and with salivary gland homogenate of *P. perniciosus* (Per-SGH) as depicted on the scheme **(B)**. Phosphate-buffered saline (PBS) was used as a negative control. The representative picture of the two independent experiments is shown **(A)**.

Additionally, we tested the ability of rSP03 to elicit delayed-type hypersensitivity (DTH) in the intradermal skin test and compared it with the second yellow-related protein rSP03B and Per-SGH. Neither rSP03, rSP03B, nor the PBS control elicited DTH reaction in the skin of mice pre-exposed to *P. perniciosus* bites measured as the visible redness in the area of inoculation (Figure 3A). On the other hand, the site of crude Per-SGH inoculation showed induration and erythema 4.9 and 6.7 mm in diameter per mouse, respectively (Figure 3).

## Discussion

In this study, we have tested three recombinant salivary proteins from *P. perniciosus* females for their effect on the host immunity. Those recombinant proteins were the apyrase rSP01, and the two proteins of the yellow-related family, rSP03 and rSP03B. We tested their function, focusing on the macrophages as the key host cells for *Leishmania* parasites. Macrophages are known to respond to the different stimuli in their surrounding microenvironment by expressing arginine-processing enzymes, an iNOS or an arginase, whose products are either leishmanicidal (nitric oxide) or essential for *Leishmania* survival (L-ornithine and its metabolites), respectively (Tomiotto-Pellissier et al., 2018). Here we showed that the recombinant *P. perniciosus* apyrase and yellow related proteins possess anti-inflammatory properties on murine macrophages.

Apyrases are present in saliva of all sand fly species studied to date (Oliveira et al., 2020; Polanska et al., 2020). This enzyme hydrolyzes ADP and ATP, thus destroying the important stimulus of platelet aggregation and inflammation at the bite site, which helps the female fly to complete the bloodmeal (Francischetti, 2011). So far, the sand fly apyrases have been tested only in the context of adaptive immunity, employing transfection of the host's cells (Oliveira et al., 2006, 2008; Gomes et al., 2008; Xu et al., 2011; Marzouki et al., 2012; Tlili et al., 2018; Gholami et al., 2019). In previous studies, two recombinant *P. perniciosus* salivary apyrases, rSP01 and rSP01B, have been prepared, however, they have been primarily tested as the markers of exposure with sera of bitten hosts (Martin-Martin et al., 2013, 2014, 2015; Drahota et al., 2014; Kostalova et al., 2015) and never subjected to test their immunomodulatory properties. Here, we present the first study showing immunomodulatory effect of the recombinant sand fly apyrase on the macrophage activity *in vitro*. In our study, *P. perniciosus* rSP01 significantly inhibited NO production in murine macrophages. The inhibition was pronounced in all three macrophage populations, including the macrophages stimulated toward the pro-inflammatory state, suggesting an anti-inflammatory nature of this recombinant protein.

Yellow-related proteins (YRPs) comprise highly abundant proteins in sand fly saliva. They serve as the binders of biogenic amines (such as serotonin and norepinephrine) thus supporting successful blood feeding by scavenging important stimuli of haemostatic reactions at the bite site (Xu et al., 2011; Sumova et al., 2019). In *P. perniciosus*, two YRPs have been identified and named PpeSP03 and PpeSP03B (Anderson et al., 2006). Although sequentially similar, they differ in several aspects. The PpeSP03B protein has alkaline pI, is more abundant, and serves as a high-affinity binder of serotonin (Anderson et al., 2006; Martin-Martin et al., 2012; Sumova et al., 2019) and a strong binder of anti-saliva antibodies in mice and dogs (Anderson et al., 2006; Vlkova et al., 2011; Martin-Martin et al., 2012; Drahota et al., 2014; Kostalova et al., 2015; Willen et al., 2019). The latter properties of rSP03B made it the best antigen to substitute the sand fly salivary gland homogenate in assays measuring anti-*P. perniciosus* saliva IgG antibodies (Kostalova et al., 2015, 2017), upgraded later into the immunochromatographic test to screen dogs in endemic areas for the vector exposure (Willen et al., 2018, 2019). The other *P. perniciosus* YRP, PpeSP03, has lower pI, is less abundant, and acts as a medium affinity binder of norepinephrine (Anderson et al., 2006; Martin-Martin et al., 2012; Sumova et al., 2019) and a weak binder of anti-saliva antibodies in naturally-exposed dogs (Vlkova et al., 2011; Kostalova et al., 2017).

In our study, both *P. perniciosus* recombinant YRPs showed different immunomodulatory activity but with overall similar anti-inflammatory effect. We demonstrated that the recombinant protein rSP03B acted similar to the *P. perniciosus* rSP01 apyrase; it significantly inhibited NO production in all three murine macrophage populations, while showing no effect on the macrophage arginase activity. The other YRP, rSP03, showed the most pronounced effect on murine macrophages among the three proteins tested. This protein decreased the NO production in stimulated macrophages and increased the arginase activity along with a strong stimulation of IL-10 production in all types of macrophages tested. This anti-inflammatory effect of rSP03 was also seen in macrophages stimulated toward the pro-inflammatory phenotype.

Additionally, we also tested both YRPs for their effect on the adaptive immunity. In mice pre-exposed to *P. perniciosus* bites, injection of the recombinant YRPs did not elicit visible skin reaction compared to *P. perniciosus* crude saliva. This result is in accordance with measured anti-inflammatory effect of both yellow-related proteins. Similar tests have been performed with the YRPs from other sand fly species, employing a different experimental approach; mice were immunized intradermally with a plasmid coding for sand fly YRP and challenged with saliva from the relevant sand fly species (and vice versa; mice first exposed to sand fly saliva and then challenged with plasmid). In these settings, ten YRPs from five different sand fly species were tested with different outcomes (Anderson et al., 2006; Gomes et al., 2008; Oliveira et al., 2008, 2015; Collin et al., 2009; Xu et al., 2011; Abbehusen et al., 2018; Gholami et al., 2019). For instance, in well-studied *Lutzomyia longipalpis*, only one of the three YRPs showed a positive recall response, while the other two proteins did not elicit any DTH response in mice (Gomes et al., 2008; Xu et al., 2011). Such functional divergence of the YRPs is in accordance with the gene duplication events in this protein family (Coutinho-Abreu and Valenzuela, 2018) providing most of the sand fly species with multiple YRPs with various function for scavenging biomolecules essential for host haemostasis and for modulating the host immune system (Sumova et al., 2019).

## Conclusion

**Figure 4:**
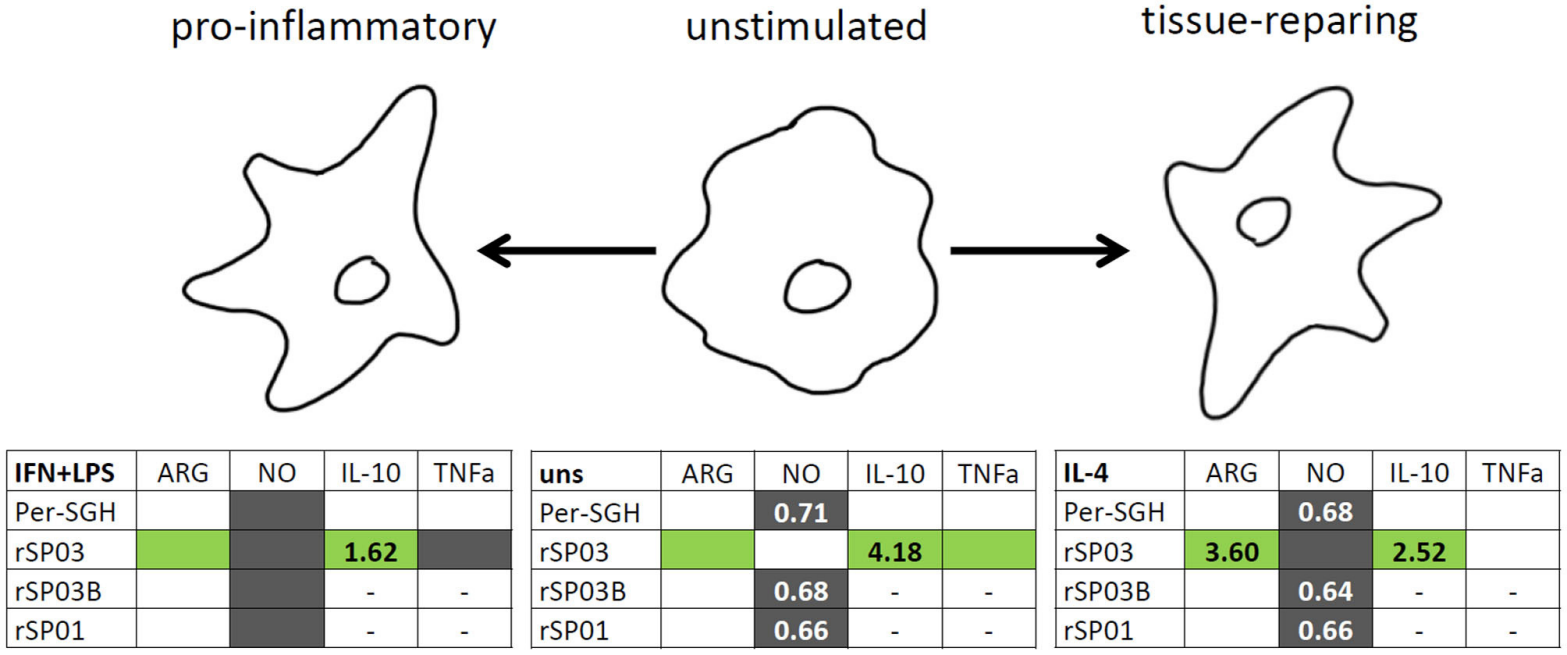
Graphical summary of the effect of *Phlebotomus perniciosus* salivary proteins on macrophages. Bone marrow-derived macrophages of BALB/c mice were incubated with salivary gland homogenate of *Phlebotomus perniciosus* (Per-SGH), or with one of the three *P. perniciosus* recombinant proteins: Yellow-related proteins rSP03 and rSP03B, or Apyrase rSP01. The macrophages were stimulated with either IL-4 (resulting in tissue-repairing phenotype), a combination of IFN-γ and bacterial lipopolysaccharide (resulting in pro-inflammatory phenotype), or left unstimulated (uns). The experiment was performed in six biological replicates (6 mice). Within the tables, 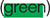 denotes significant increase and 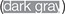 significant decrease in the production of urea (ARG), nitrite (NO), IL-10 and TNF-α cytokines. Insignificant changes (*p* > 0.05) are marked (white), (–) means not measured. The numbers represent fold changes above 20% compared to the control.

In summary, all three recombinant *P. perniciosus* proteins showed anti-inflammatory properties on murine macrophages (Figure 4). Even in the presence of pro-inflammatory stimuli (IFN-γ and LPS), all tested recombinants inhibited NO production. Moreover, rSP03 seems to have a very strong anti-inflammatory effect since it enhanced arginase activity, increased the production of IL-10, and inhibited the production of TNF-α even in macrophages stimulated with IFN-γ and LPS. These results also suggest that *P. perniciosus* apyrase and yellow-related proteins may serve as enhancing factors in sand fly saliva, facilitating the development of *Leishmania* infection along with their anti-haemostatic functions.

In general, sand fly salivary proteins in a recombinant form might be used to understand the possible function of their native counterparts. Importantly, if the recombinant proteins are tested as candidates for leishmaniasis vaccine (Collin et al., 2009; Gomes et al., 2012; Abbehusen et al., 2018; Cunha et al., 2018), their possible immunogenic nature should be also considered since it may negatively influence the vaccinated individuals (Reagan et al., 2012), e.g., by initiating an autoimmune disease in susceptible recipients (Qian et al., 2016). Thus, any vaccine candidate molecules should be thoroughly tested to validate their overall beneficial status for the host immunity.

## Data Availability Statement

The raw data supporting the conclusions of this article will be made available by the authors, without undue reservation.

## Ethics Statement

The animal study was reviewed and approved by The Committee on Ethics of Laboratory Experiments, Faculty of Science, Charles University, Czech Republic.

## Author Contributions

IR and PV conceived the study. IR, PV, PS, and NP designed the experiments. PS and NP performed the experiments. BK expressed and purified the recombinant proteins. TL maintained the *P. perniciosus* colony and designed the methodology of BMMF macrophage cultivation. TS performed statistical analysis. IR analyzed and interpreted the data and drafted the manuscript. PS and NP contributed to the draft writing. OV and PV provided reagents and material and critically read the manuscript. All the authors read and approved the final version of the manuscript.

## Conflict of Interest

The authors declare that the research was conducted in the absence of any commercial or financial relationships that could be construed as a potential conflict of interest.
